# A “Significant” Shift in Statistical Interpretation in *Ecology and Evolution*: A Helpful Guide for Authors and Reviewers

**DOI:** 10.1002/ece3.74659

**Published:** 2026-11-08

**Authors:** Jason T. Fisher

**Affiliations:** ^1^ Senior Editor, Ecology and Evolution Victoria Canada; ^2^ School of Environmental Studies University of Victoria Victoria British Columbia Canada

## Abstract

“Statistical significance”, wherein the deviation of some measure of a sample population is greater than an expected threshold (usually *p* > 0.05) is a concept and practice strongly embedded in the life sciences. However, the use of “statistical significance” has been deprecated by the American Statistical Association (ASA), and that news has been slow to spread across disciplines. The result of this misuse is that many authors are wrongfully claiming “no effect” of a variable when *p* > 0.05 or confidence intervals (CIs) overlap zero. To ensure scientific rigor in *Ecology and Evolution*, we offer this brief guide to authors and reviewers and strongly encourage authors to use the ASA statement on *p*‐values. The ASA suggests that authors report effect sizes and attendant error in ways that let users judge support for the hypotheses represented in a model, avoiding the binary “true or false” of *p* = 0.05 or overlapping CIs. Furthermore, we encourage authors to acquaint themselves with numerous other common mistakes in statistical interpretation highlighted by the ASA and subsequent authors. By incorporating these principles into statistical reporting and results, information and patterns that might otherwise be overlooked remain available for readers to evaluate. We recognize that changes in reporting practices will not occur overnight, so our aim is to help authors adopt ASA principles: to that end we describe potential problems and offer guidelines for authors and reviewers to follow, to avoid incorrect statistical interpretations and spurious conclusions.

## Abstract 2

Statistical significance is a concept and practice that has been deprecated by the American Association but that news is slow to spread across disciplines. In *Ecology and Evolution*, we recommend that authors eschew alpha values for *p* as a threshold for support for hypotheses. Instead, following publishing guidelines and advice, authors should report effect sizes and attendant error in ways that let users judge support for the hypotheses represented in a model, consistent with a weight‐of‐evidence approach.

## 1 Introduction

*Ecology and evolution* are in large part empirical, quantitative disciplines, following theory and practice established by mathematicians and statisticians (Fisher 1958; Zar 1974). The idea of *statistical significance* introduced by R.A. Fisher (1925) and expounded by Neyman and Pearson (1933) (nicely reviewed by Hurlbert and Lombardi (2009)) is now deeply enculturated in ecological modeling, with the alpha value of *p* = 0.05 the common arbiter of the outcome of a statistical analysis: *true* or *false*, *significant* or *insignificant*. However, *statistical significance* has been known to be problematic for some time. Johnson (1999)'s paper *The Insignificance of Statistical Significance Testing* drained the power of null‐hypothesis testing (NHT) from wildlife sciences and started a paradigm shift toward other more robust methods. This paper started a revolution with a general replacement of NHT by multiple model selection based on information theory (Akaike 1973; Burnham and Anderson 2002). Nonetheless, *p* values remain a common and useful tool for judging how an observed sample mean deviates from a second sample, or a theoretical population mean. *p* values remain useful, but we encourage *Ecology and Evolution* authors to ensure their use of *p* values is free of misinterpretation. The purpose of this editorial is to provide a brief, hopefully helpful and informative guide alerting authors, reviewers, and readers to this body of literature.

Wasserstein and Lazar (2016) outline the principal arguments in the ASA statement (direct quotes in italics):

1. *p‐values can indicate how incompatible the data are with a specified statistical model*.
2. *p‐values do not measure the probability that the studied hypothesis is true, or the probability that the data were produced by random chance alone*.
3. *Scientific conclusions and business or policy decisions should not be based only on whether a p‐value passes a specific threshold*. This is a key point worth elaborating: there is no mathematical or logical basis for a hardline demarcation between “true” and “false”. Instead, conclusions are made by weighing probabilities thoughtfully and carefully, armed with appropriate information, leading to the next point.
4. *Proper inference requires full reporting and transparency*. Do not report only “significant” *p*‐values: report all *p*‐values—along with the appropriate effect sizes, sample sizes, and attendant error—and let the reader judge.
5. *A p‐value, or statistical significance, does not measure the size of an effect or the importance of a result*. Statistical significance is not equatable to scientific significance, nor other measures of impact to which the analysis pertains. For example, a regression between variable *X* and *Y* yielding a regression slope of 0.01 with a *p* = 0.002 is not a stronger relationship than a regression slope of 1.0 with a *p* = 0.07. The effect of *X* on *Y* is 100 times greater in the second regression, even though the probability of departure from the assumed distribution is lower.
6. *By itself, a p‐value does not provide a good measure of evidence regarding a model or hypothesis*. This is where information theory, model validation, and other diagnostics yield valuable additional information about the statistical support for a hypothesis.

This is a substantial departure from what most of us were, or are, being taught in introductory statistics sources in the life sciences, where *p* = 0.05 is the arbiter of truth. If *p* does not measure the probability the null hypothesis is true (or conversely, the probability that the alternative hypothesis is false), what does it measure? Here Greenland et al. (2016) elaborate very effectively. *Specifically, the distance between the data and the model prediction is measured using a test statistic (such as a t‐statistic or a Chi squared statistic). The p value is then the probability that the chosen test statistic would have been at least as large as its observed value if every model assumption were correct, including the test hypothesis*. (In the original formulation, Fisher (1925) elaborates that this probability emerges if the experiment is repeated identically 1000s of times, a critical piece of perspective that seems to have been lost). This again is a key point: *p* values test all assumptions about data generation, not the targeted (or null) hypothesis. True, a small *p* value indicates a marked departure from the assumed data distribution, but it does not inform where that departure occurs: in the effect size (which is commonly concluded), the sample size, the study design, the model construct, or model assumptions and violations. In fact, the *p* value tells us nothing about the hypothesis unless we are fully certain that all other assumptions are fully and repeatably met. Even then, a *p*‐value is only useful as *a continuous measure of the compatibility between the data and the entire model used to compute it, ranging from 0 for complete incompatibility to 1 for perfect compatibility* (Greenland et al. 2016). A recent study published in *Ecology and Evolution* by Arno et al. (2026) demonstrates that statistical significance is not equivalent to evidential support, showing that *p*‐values can provide a misleading picture of the strength of evidence, particularly in the complex analyses common to *ecology and evolution*. Their work highlights the need for ecologists to move beyond sole reliance on null‐hypothesis significance testing and adopt complementary approaches that more directly quantify support for competing hypotheses.

*p*‐values are not alone in having their misuse firmly enculturated in the fields of *ecology and evolution*: confidence intervals (CI) are also subject to the same problem. The CIs of two population means overlapping is often viewed as “these two populations/measures do not differ”. Likewise, a CI overlapping zero is used as a threshold for “no effect” or “this population/measure is not different from zero”. Neither are correct. More specifically, these interpretations are false:

1. *The specific 95% confidence interval presented by a study has a 95% chance of containing the true effect size*.
2. *An effect size outside the 95% confidence interval has been refuted (or excluded) by the data.*
3. *If two confidence intervals overlap, the difference between two estimates or studies is not significant*.
4. *An observed 95% confidence interval predicts that 95% of the estimates from future studies will fall inside the observed interval*.
5. *If one 95% confidence interval includes the null value and another excludes that value, the interval excluding the null is the more precise one* (Greenland et al. 2016).

More guidance is provided by Schenker and Gentleman (2001). As with *p*‐values, CIs are outcomes of the test statistic, the sampling size, and sampling design, the model, and the model assumptions. They are best used as a scale with which to weigh the strength of evidence, not as an arbiter of truth at some threshold limit.

## 2 Discussion

ASA's statement on statistical significance has been very slow to take hold in the broader scientific literature (Ferreira et al. 2026). Berner and Amrhein (2022) outline extensive (mis)use of *p*‐values in the journal *Evolutionary Biology*. My search within Wiley's journals^1^ shows 867,449 uses of the term *statistically significant* since 2020; 8124 times in *Ecology and Evolution* alone. Even in recent articles providing statistical advice, the common misinterpretations perpetuate: in *Writing Statistical Methods for Ecologists*, Davis and Kay (2023) specifically advocate for articulating a *p* = 0.05 (or alternative) as a “cutoff”. Gilbert et al. (2024) in *A Century of Statistical Ecology* reference “significance” in multiple applications.

Ergo, we are not alone in this slow change. In *The ASA's p‐Value Statement: One Year On*, Matthews et al. (2017) note how little has shifted in that time; statistical significance is “too familiar to ditch”. Other statisticians have joined in: Hurlbert et al. (2019) proclaim a “*coup de grace* for a tough old bird, as statistical significance expires”. Eight hundred scientists have since signed onto the Commentary published in *Nature*, *Retire statistical significance* (Amrhein et al. 2019a), highlighted in the follow‐up article *Scientists Rise Up Against Statistical Significance* (Amrhein et al. 2019b).

### 2.1 Incorporating These Principles Into Your Manuscript

So how do we proceed in a post‐significance world? The advice from ASA's executive and leading statisticians mostly centers around breaking old habits, with plenty of “Don'ts”. “Do's”, they admit, are harder to come by as a new paradigm has yet to emerge to fully replace the old. However, it may be the point that a new paradigm is not needed, merely a more widespread acceptance that there is no hard line between truth and falsity, because these nebulous constructs are unobservable. Instead, we need to adopt more comfort with uncertainty and responsibility for being our own judge. In *Moving to a World Beyond “p < 0.05”*, Wasserstein et al. (2019) advocate for ATOM: Accept uncertainty; be thoughtful, open, and modest.

1. Report and comment on effect sizes, as well as attendant estimated error. Likewise, Discussion should be focused on effect sizes primarily, and attendant error estimate secondarily.
2. Reject the use of an alpha value (*p* = 0.05, or other) or overlapping CIs as a measure of the value of a modeling outcome; report all *p* values, *N*, and means (or effect sizes).
3. Use language about the “strength of evidence” and let the reader judge, rather than a threshold for “statistical significance”.
4. Be thoughtful and critical about the choice of model construct. State all model assumptions and provide an honest appraisal of how the model met, or deviated from, those assumptions.
5. Probe how design decisions may have influenced the outcome. Run sensitivity tests. Satisfy yourself that the results hold across a range of analytical decisions.

### 2.2 Key Steps for Getting Started

1. Read the ASA statement on *p*‐values (Wasserstein and Lazar 2016).
2. Read Greenland et al. (2016); they list 18 common misinterpretations and misuses of *p*‐values, and another 5 misuses of confidence intervals.
3. If you have made one of these mistakes, follow their advice and that of Wasserstein and Lazar (2016), Wasserstein et al. (2019), and Anderson (2019) to adopt a correct interpretation.

## 3 Conclusions

Statistical models in all their forms can be invaluable tools for understanding nature and the differences between its constituent elements. Cribbing Box (1976)'s aphorism, “All models are wrong; some models are useful”; like any tool, utility depends on the appropriateness and skill of application. We encourage authors, reviewers, and readers at *Ecology and Evolution* to become familiar with the ASA Statement and the associated papers, which offer excellent advice on how to interpret effect sizes and estimated error from statistical models. Ultimately there is no arbiter of “significant” or “non‐significant”, true or false. There are instead multiple pieces of evidence that must be weighed quantitatively and qualitatively to inform a conclusion, and we encourage scientists engaging with *Ecology and Evolution* to explore that evidence thoroughly. While we recognize that widespread changes in statistical reporting will take time, we want authors to know that we strongly support their use. We believe they improve the reporting of data and contribute to a more transparent, informative, and meaningful communication of scientific results.

## Author Contributions

**Jason T. Fisher:** conceptualization (lead), investigation (lead), project administration (lead), resources (lead), writing – original draft (lead), writing – review and editing (lead).

## Conflicts of Interest

The author declares no conflicts of interest.

## Data Availability

No data were used in this editorial.

## Endnotes

^1^

https://onlinelibrary.wiley.com/action/doSearch?field1=AllField&text1=statistically+significant&publication=&Ppub=&AfterMonth=1&AfterYear=2020&BeforeMonth=5&BeforeYear=2026&content=articlesChapters&target=default.
